# Supplementary material for: SIRT7 Is a Lysine Deacylase with a Preference for Depropionylation and Demyristoylation
Source: Int J Mol Sci. 2025 Mar 28;26(7):3153. doi: 10.3390/ijms26073153 (PMC11988671; doi:10.3390/ijms26073153)
Supplement: Supplementary file 1 [file ijms-26-03153-s001.zip › ijms-3539083-supplementary.pdf]

International Journal of *Molecular Sciences*

MDPI

Supplementary Materials:

SIRT7 is a lysine deacylase with a preference for depropionylation and demyristoylation

Mohammad Golam Kibria[1], Tatsuya Yoshizawa[1,2,*], Tianli Zhang[3], Katsuhiko Ono[4], Tomoya Mizumoto[1], Yoshifumi Sato[1], Tomohiro Sawa[4], Kazuya Yamagata[1,5,*]

[1] Department of Medical Biochemistry, Faculty of Life Sciences, Kumamoto University, Kumamoto 860-8556, Japan; kibria.bmb@gmail.com (M.G.K.); mizumoto-t@kumamoto-u.ac.jp (T.M.); ysato413@kumamoto-u.ac.jp (Y.S.); k-yamaga@kumamoto-u.ac.jp (K.Y.)
[2] Cell Biology, Graduate School of Medical Science, Kyoto Prefectural University of Medicine, Kyoto 606-0823, Japan; yoshizaw@koto.kpu-m.ac.jp (T.Y.)
[3] Center for Integrated Control, Epidemiology and Molecular Pathophysiology of Infectious Diseases, Akita University, Akita 010-8543, Japan; zhangt@med.akita-u.ac.jp (T.Z.)
[4] Department of Microbiology, Faculty of Life Sciences, Kumamoto University, Kumamoto 860-8556, Japan; sawat@kumamoto-u.ac.jp (T.S.); onokat@kumamoto-u.ac.jp (K.O.)
[5] Center for Metabolic Regulation of Healthy Aging (CMHA), Faculty of Life Sciences, Kumamoto University, Kumamoto 860-8556, Japan; k-yamaga@kumamoto-u.ac.jp (K.Y.)
* Correspondence: k-yamaga@kumamoto-u.ac.jp (K.Y.); yoshizaw@koto.kpu-m.ac.jp (T.Y.); Tel.: +81-96-373-5068 (K.Y.); Tel.: +81-75-703-4940 (T.Y.)

Contents:

Supplementary Figure S1. Supplementary data for SIRT1 deacetylation activity.

Supplementary Figure S2. Supplementary data for NAD^+ levels in Hepa1-6 cells.

Supplementary Table S1. MRM Parameters for analytes.

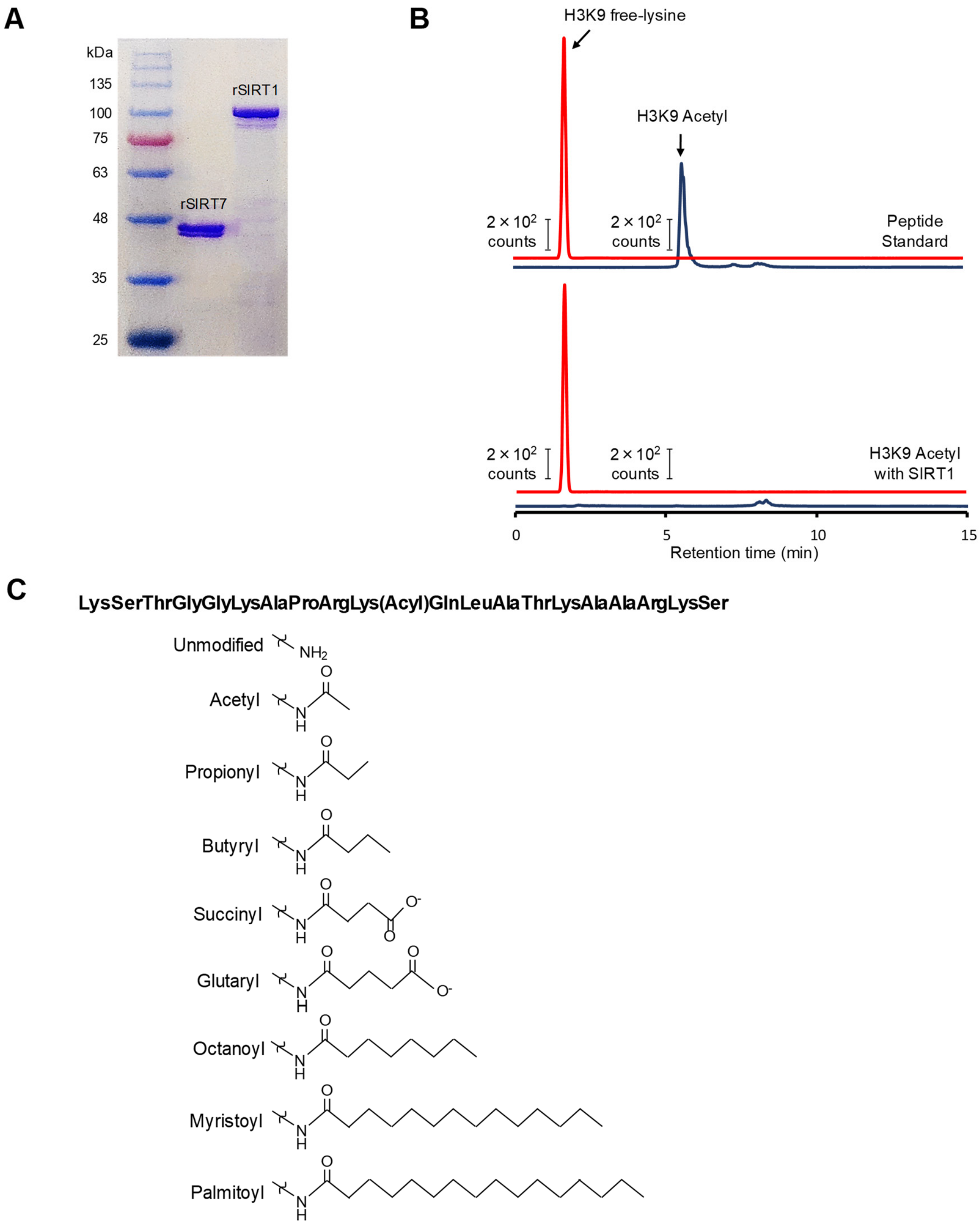

Figure S1. Supplementary data for SIRT1 deacetylation activity. (**A**) Coomassie brilliant blue staining of recombinant SIRT7 and SIRT1 purified from *E. coli*. (**B**) *In vitro* deacylation activity of SIRT1 analyzed by LC-MS/MS. (**C**) Synthetic peptide corresponding to the region of histone H3 containing the acyl-modified lysine 18 used for the SIRT7 deacylation assay.

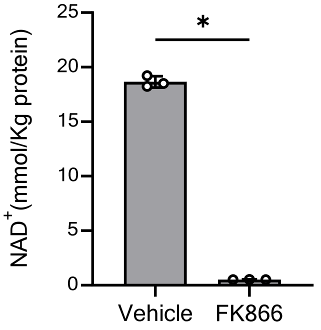

Figure S2. Supplementary data for NAD^+ levels in Hepa1-6 cells. Cellular NAD^+ levels in Hepa1-6 cells treated with 8 nM FK866. The data are presented as the mean ± SD of three independent experiments. Two-tailed Student's *t*-test. $^*p < 0.05$.

Table S1. MRM Parameters for analytes.

Analytes	Precursor charge state	Precursor Ion (*m*/*z*)	Product Ion (*m*/*z*)	Fragmentor Voltage (V)	Collision Energy (eV)	Polarity
H3K18 Acetyl	$[M+4H]^{4+}$	426.3	84.1	90	55	positive
H3K18 Propionyl	$[M+4H]^{4+}$	536.1	84.2	130	55	positive
H3K18 Butyryl	$[M+2H]^{2+}$	1078.3	1025.6	50	49	positive
H3K18 Succinyl	$[M+4H]^{4+}$	547.1	84.2	130	55	positive
H3K18 Glutaryl	$[M+3H]^{3+}$	738.9	84.2	50	55	positive
H3K18 Octanoyl	$[M+4H]^{4+}$	553.7	554.2	130	9	positive
H3K18 Myristoyl	$[M+2H]^{2+}$	1148.4	84.1	50	55	positive
H3K18 Palmitoyl	$[M+4H]^{4+}$	581.7	582.2	130	9	positive
H3K18 free-lysine	$[M+4H]^{4+}$	417.8	418.2	90	55	positive
H3K9 free-lysine	$[M+4H]^{4+}$	572.7	84.2	130	55	positive
H3K9 Acetyl	$[M+4H]^{4+}$	583.2	70.3	130	55	positive
